# Multi-omics analysis on the pathogenicity of *Enterobacter cloacae* ENHKU01 isolated from sewage outfalls along the Ningbo coastline

**DOI:** 10.1186/s12953-016-0104-y

**Published:** 2016-10-18

**Authors:** Dijun Zhang, Weina He, Qianqian Tong, Jun Zhou, Xiurong Su

**Affiliations:** School of Marine Science, Ningbo University, 818 Fenghua Road, Ningbo, Zhejiang Province 315211 People’s Republic of China

**Keywords:** *Enterobacter cloacae*, Pathogenicity, Iron, Proteomic, Metabolomic

## Abstract

**Background:**

The acquisition of iron is important for the pathogenicity of bacteria and blood. Three different culture environments (Fe stimulation, blood agar plate and normal plate) were used to stimulate *Enterobacter cloacae*, and their respective pathogenicities were compared at the proteomic, mRNA and metabolomic levels.

**Methods:**

2D-DIGE combined with MALDI-TOF-MS/MS, RT-PCR and ^1^H NMR were used to analyze the differential expression levels of proteins, mRNA and metabolites.

**Results:**

A total of 109 proteins were identified by 2D-DIGE and mass spectrometry after pairwise comparison within three culture environments, clustered into 3 classes and 183 functional categories, which were involved in 23 pathways. Based on the 2D-DIGE results, multiple proteins were selected for verification by mRNA expression. These results confirmed that most of the proteins were regulated at the transcriptional level. Thirty-eight metabolites were detected by NMR, which correlated with the differentially expressed proteins under different treatment conditions.

**Conclusions:**

The results show that culture in a blood agar plate and a suitable concentration of iron promote the pathogenicity of *E. cloacae* and that high iron concentrations may have adverse effects on growth and iron uptake and utilization by *E. cloacae*.

**Electronic supplementary material:**

The online version of this article (doi:10.1186/s12953-016-0104-y) contains supplementary material, which is available to authorized users.

## Background

Due to antibiotics that have been overly prescribed in recent years, *Enterobacter cloacae* has emerged as an important nosocomial pathogen in neonatal units, with numerous outbreaks of infection being reported [1, 2]. *E. cloacae* occur in water, sewage, soil, food, and as commensal microflora in the intestinal tracts of humans and animals [3]. Molecular biological studies of *E. cloacae* have revealed six species, and some strains that have been phenotypically identified as *E. cloacae* are opportunistic pathogens that have been implicated as the causative agent of local and systemic infections in humans [4]. They are important nosocomial pathogens that are responsible for bacteremia, lower respiratory tract, skin, soft tissue, urinary tract, intra-abdominal and ophthalmic infections, endocarditis, septic arthritis and osteomyelitis, especially the outbreaks of septicemia in the neonatal intensive care unit [5, 6]. This bacterium may be transmitted to neonates through intravenous fluids, total parenteral nutrition solutions and medical equipment. Common endogenous reservoirs of *E. cloacae* include the gastrointestinal tract of healthy adults and the urinary and respiratory tracts of sick patients. Sputum, secretions and pus, and urine are the most studied specimens of human *E. cloacae* infection [7].

*E. cloacae* is isolated from the feces of 10–70 % of neonates. Due to their relative lack of toxicity and ability to cross the blood–brain barrier, these antimicrobial agents have been increasingly used as first-line antibiotic therapy in neonates. As a result, *E. cloacae* has become super-bacteria in hospitals due to the presence of extended-spectrum β-lactamases (ESBLs) [1]. Although *E. cloacae* complex strains are among the most common *Enterobacter* species causing nosocomial bloodstream infections in the last decade, little is known regarding their virulence-associated properties. Among the most common risk factors for developing *E. cloacae* bloodstream infections are prolonged hospitalization, the severity of the illness, and exposure to invasive procedures [4]. Additional predisposing factors are the usage of a central venous catheter, prolonged antibiotic therapy, parenteral nutrition and immunosuppressive therapy [8].

In our previous study, we obtained 98 strains of *E. cloacae* from the Ningbo sewage outfall using *rpo*B genotyping, multi-locus sequence analysis and comparative genomic hybridization. Among the 98 strains of bacteria, the following virulence genes were identified: iron regulatory protein 2 (*irp*2), ferrichrome-iron uptake receptor (*fhu*A), superoxide dismutase B (*sod*B), and Shiga-Like-Toxin A (*slt*A), with a detection rate of 35.71 % for the *fhu*A^+^ *irp*2^+^ *sod*B^+^ genotype, 25.27 % for *fhu*A^+^ *irp*2^+^ *slt*A^+^, 13.19 % for *irp*2^+^, 12.09 % for *fhu*A^+^, 9.89 % for *fhu*A^+^ *irp*2^+^, 8.79 % for *slt*A^+^ *sod*B^+^, and 8.79 % for *fhu*A^+^ *irp*2^+^ *sod*B^+^ *slt*A^+^ [9].

The ability of bacteria to acquire iron from the external environment is known to have a strong relationship with virulence [10, 11]. Iron is an essential element for most bacteria; it is utilized as the reaction center for redox enzymes and directly participates in redox reactions by switching between the Fe^2+^ and Fe^3+^ states [12]. Among the Gram-positive pathogens, iron uptake in *Staphylococcus aureus* has been investigated most extensively [13]. In a study of Gram-negative bacteria isolated from 120 neonate blood samples with clinical signs of infection, *E. cloacae* accounted for the largest population among the pathogenic bacteria [14]. The blood agar plate is one of the most important methods for cultivating *E. cloacae* and other pathogenic bacteria to study their pathogenicity [15]. Therefore, to study the pathogenicity of *E. cloacae* isolated from sewage outfall, we set out to compare the regulation of *E. cloacae* pathogenicity by blood and iron availability. We cultured *E. cloacae* in three different media, and then assessed pathogenicity by 2D-DIGE, RT-PCR and nuclear magnetic resonance (NMR) at proteomic, mRNA and metabolic levels.

## Methods

### Isolation, identification and culture of bacteria

*E. cloacae* was isolated from sewage outfalls along the Ningbo coastline (Ningbo, China) and positively identified as *Enterobacter cloacae* ENHKU01 by sequencing using universal primers (27 F: 5’-AGAGTTTGATCCTGGCTCAG-3’ and 1492R: 5’-GGTTACCTTGTTACGACTT-3’). *E. cloacae* was cultured on blood agar plates in the first experimental group (hereafter referred to as Y1) and in beef extract peptone medium (5 mg/mL beef extract powder, 10 mg/mL peptone, 20 mg/mL agar, all purchased from Microbial Reagent, Hangzhou, China) in the control group (hereafter referred to as Y2). In the second experimental group (hereafter referred to as Y3), 0.1 mM FeCl_3_ (this concentration was selected from a preliminary experiment with varying concentrations of Fe^3+^, Additional file 1) was added to the same medium for 12 h at 28 °C. All extractions and experiments were performed in a cold room at 4 °C. *E. cloacae* were washed twice with phosphate-buffered saline (PBS), and the bacteria were collected after centrifugation (6,000 rpm, 15 min, 4 °C).

### Protein identification

#### Sample preparation and CyDye labeling

The bacteria were dissolved in 10 mL of lysis buffer (8 mol/L urea, 2 mol/L thiourea, 4 % (w/v) CHAPS, 10 mg/mL of DTT, 2.5 mg/mL of Tris), and protein was subsequently extracted by ultrasonic disruption (200 W for 10 min) on ice. Centrifugation (12,000 rpm, 30 min, 4 °C) was used to pellet the cell debris, and the supernatant was mixed with 5 times its volume of acetone (containing 10 % TCA). The proteins were precipitated for 6 h at −20 °C, and the supernatant discarded after centrifugation (12,000 rpm, 30 min, 4 °C). The pellet was resuspended in acetone and centrifuged (12,000 rpm, 30 min, 4 °C), and the precipitate was dried in a draft cupboard. The protein pellet was resuspended in rehydration buffer (8 mol/L urea, 2 mol/L thiourea, 40 mg/mL CHAPS, 10 mg/mL of DTT). Finally, the protein concentration was determined using a 2-D Quant Kit (Amersham Biosciences, USA) with BCA (2 mg/mL) as the standard. The optimal concentration of the protein sample was between 5 and 10 mg/mL.

For each sample, 30 μg of protein was mixed with 1.0 μl of diluted CyDye (1:5 diluted with dimethyl formamide from a 1 nmol/μl stock) and maintained in the dark on ice for 30 min. Samples from each pair were labeled with Cy3 and Cy5, respectively, while the same amount of the pooled standard containing equal quantities of all samples was labeled with Cy2 (Table 1). The three labelled and quenched samples were combined, and a total of 150 μg of protein was mixed and added to the rehydration buffer and 0.5 % Immobilized pH gradient (IPG) buffer (GE Healthcare, USA) to a final volume of 460 μL.

**Table 1:** DIGE experimental design for sample protein labeling from different treatments and internal standard

Gel No.	Cy2	Cy3	Cy5
Gel 1	Y1 + Y2 + Y3	Y1	Y2
Gel 2	Y1 + Y2 + Y3	Y2	Y1
Gel 3	Y1 + Y2 + Y3	Y3	Y2
Gel 4	Y1 + Y2 + Y3	Y1	Y3
Gel 5	Y1 + Y2 + Y3	Y3	-

#### Two-dimensional gel electrophoresis

After loading the labeled samples onto 22-cm pH 4–7 linear IPG strips (GE Healthcare, USA), iso-electric focusing (IEF) was performed as follows: 12 h of rehydration at 20 °C, followed by 300 V for 45 min, 700 V for 45 min, 1,500 V for 1.5 h, 9,000 V for 27,000 VHr, and 9,000 V for 36,000 VHr. After IEF, the IPG strips were equilibrated for sodium dodecyl sulfate-polyacrylamide gel electrophoresis (SDS-PAGE) in 5 mL equilibration buffer (0.05 M Tris–HCl (pH 8.8), 6 M urea, 30 % (v/v) glycerol, 2 % (w/v) SDS and a trace amount of bromophenol blue) containing 1 % DTT for 15 min, followed by a second equilibration step of 15 min with the same buffer containing 2.5 % (w/v) iodoacetamide. The equilibrated strips were loaded on the top of 12 % SDS-polyacrylamide gels and sealed with 0.5 % (w/v) agarose. The SDS-PAGE step was performed at 15 °C in an Ettan Dalt Twelve (Amersham Biosciences, USA) electrophoresis system at 2 W/gel for 45 min, followed by 17 W/gel for approximately 4.5 h (until the bromophenol blue reached the bottom of the gel).

#### Image acquisition and analysis

The CyDye-labelled gels were visualized using a TyphoonTM 9400 imager (GE Healthcare, USA) with the appropriate excitation and emission wavelength filters for each dye, according to the manufacturer’s recommendations. All images were processed using Imagemaster 7.0 and then analyzed with DeCyder software (GE Healthcare, USA). The intra-gel analysis was performed using the DeCyder Difference In-gel Analysis system, and inter-gel matching was performed using the DeCyder Biological Variance Analysis, Statistical analyses were conducted for each sample. The spot volume ratios that showed a statistically significant (abundance variation of at least 1.5-fold, *p* < 0.05) difference were processed for further analysis.

#### Protein digestion and mass spectrometric analysis

Selected protein spots were excised from the preparative gels. Each small gel plug was destained with 100 μL of ACN in 50 mM ammonium hydrogen carbonate for approximately 1 h at room temperature, and this step was repeated until the gel was colorless. After evaporation of the solvent by vacuum centrifugation, each gel plug was rehydrated with 20 μL of 0.01 mg/mL sequencing-grade modified trypsin (Promega, Madison, WI, USA), and the mixture was agitated overnight at 37 °C. The supernatants were collected, and the gel pieces were rinsed once with 5 % TFA in 50 % ACN and then twice with 2.5 % TFA in 50 % ACN. The supernatants were then combined and lyophilized. The lyophilized peptides were dissolved in 5 mg/mL CHCA (Sigma, USA) in 50 % ACN and 0.1 % TFA. All MS/MS experiments were performed on an Autoflex speed™ MALDI-TOF-MS/MS analyzer (Bruker Daltonics, Germany). The detection conditions were as follows: UV wavelength, 355 nm; recurrence rate, 200 Hz; accelerating voltage, 20,000 V; optimal mass resolution, 1,500 Da; mass of scanning range, 700–3,200 Da. The MS data were processed by flex Analysis (Bruker Daltonics, Germany) to produce a PKL file and analyzed with the NCBI protein sequence database using BioTools (Bruker Daltonics, Germany) via the Mascot search engine.

#### Biological analysis

Gene ontology (GO) annotations were performed for the identified sequences by MS/MS using BLASTx in the NCBI database. Blast2GO software was then used to annotate the sequence hits by BLASTx (sequences with scores of E > 1e − 05 were discarded). The GO hierarchical terms of homologous genes from the Interpro protein databases were extracted to assign putative functions to the unique sequences. In addition, unique sequences with homology to enzymes involved in metabolic pathways were mapped in accordance with the Kyoto Encyclopedia of Genes and Genomes (KEGG) database. Enzyme commission (EC) numbers were acquired for unique sequences by WUBLASTx searching of the KEGG database. The EC numbers were then used to putatively map unique sequences to specific biochemical pathways.

### Confirmation of the mRNA level by RT-PCR

#### RNA extraction and cDNA synthesis

Total RNA was extracted from frozen cell pellets using the RNeasy mini RNA extraction kit (Qiagen, Germany) according to the manufacturer’s instructions. Contaminating genomic (gDNA) was removed using on-column DNaseI digestion performed using the DNaseI digestion kit (Qiagen, Germany). Elution of total RNA was performed using 50 μl of DNase/RNase-free H_2_O, and quantified with a NanoDrop 2000 UV–vis spectrophotometer (Thermo Scientific, USA).

Total RNA (4 μg) was used as a template for reverse transcriptase reactions, which were carried out in parallel with M-MuLV Reverse Transcriptase (Sangon Biotech, Shanghai, China), following the manufacturer’s instructions. Briefly, total RNA was mixed with 10 μM of random hexanucleotide primers, incubated for 5 min at 70 °C, and kept on ice for 2 min to allow hybridization. Then, RT reaction Mix (buffer 5X, 10 mM each dNTP, RNase inhibitor (20 U/μL)) and reverse transcriptase were added according to the manufacturer’s instructions. After 60 min of incubation at 42 °C, the RT enzyme was heat-inactivated at 70 °C. In each case, the total reaction volume was 20 μL.

#### RT-PCR

Target genes associated with pathogenicity were selected based on the results of the 2D-DIGE analysis. The encoded protein sequence was matched using the NCBI database. Primers used for RT-PCR were designed using Primer3 software and are listed in Additional file 2. The amplification efficiency of the primers for the target genes and the reference gene were validated using the same program.

RT-PCR assays were performed in strip tubes (Qiagen, Germany) in a Rotor-Gene 6000 Real-Time PCR machine (Corbett, Australia) following the protocol provided with SYBR® Premix Ex TaqTM II (TaKaRa, JAPAN). Each reaction consisted of four biological replicates and was conducted in 2 μL of cDNA and 18 μL reaction mixture containing 10 μL SYBR® Premix Ex TaqTM II (2X), 0.8 μL PCR forward primer (10 μM), 0.8 μL PCR reverse primer (10 μM), 2 μL template, and 6.4 μL ddH_2_O. Each amplification consisted of a denaturation step of 10 s at 95 °C, followed by 40 cycles of 15 s denaturation at 94 °C, 10 s annealing at 55 °C and elongation for 10 s at 72 °C, and then a single fluorescence measurement. Diethyl pyrocarbonate (DEPC)-treated water was used as the negative control.

### Detection of metabolites

Metabolites were extracted from the bacterial pellets by the addition of 10 ml methanol:water = 2:1, followed by cell lysis by ultrasonic disruption at 200 W for 15 min on ice and centrifugation (12,000 rpm, 10 min, 4 °C). The supernatant was collected, and the methanol was removed with a swab in the solid phase extraction cartridge. The supernatant was then stored at −80 °C, and the metabolites were freeze-dried. The samples were then transferred onto a pre-washed ultrafiltration membrane and centrifuged (6,000 rpm, 30 min, 4 °C) twice. Filtrates were collected and mixed with ACDSS (Anachro Certified DSS Standard Solution), vortexed (10 s) and centrifuged (13,000 rpm, 2 min, 4 °C).

The ^1^H NMR measurements were performed at 298 K on a Bruker Avance III 600 MHz spectrometer equipped with an inverse detection cryogenic probe (Bruker Biospin, Germany), which was operated at 600.13 MHz for a ^1^H resonance frequency. A noesypr1d/noesygppr1d pulse sequence was used to determine the bacterial metabolite profiles. One hundred twenty-eight transitions were collected as 32,768 data points for each spectrum. The ^1^H NMR signal was imported into the Chenomx NMR suite version 7.6 (Chenomx, Canada), and the data were automatically Fourier-transformed, phase-adjusted and baseline-adjusted. Metabolites from *E. cloacae* were quantified using the concentration and peak area of DSS-d6 (2,2-dimethyl-2-silapentane-5-sulfonate-d6 sodium salt) as the standard.

## Results

### Differential expression of E. cloacae proteins

**Fig. 1:**
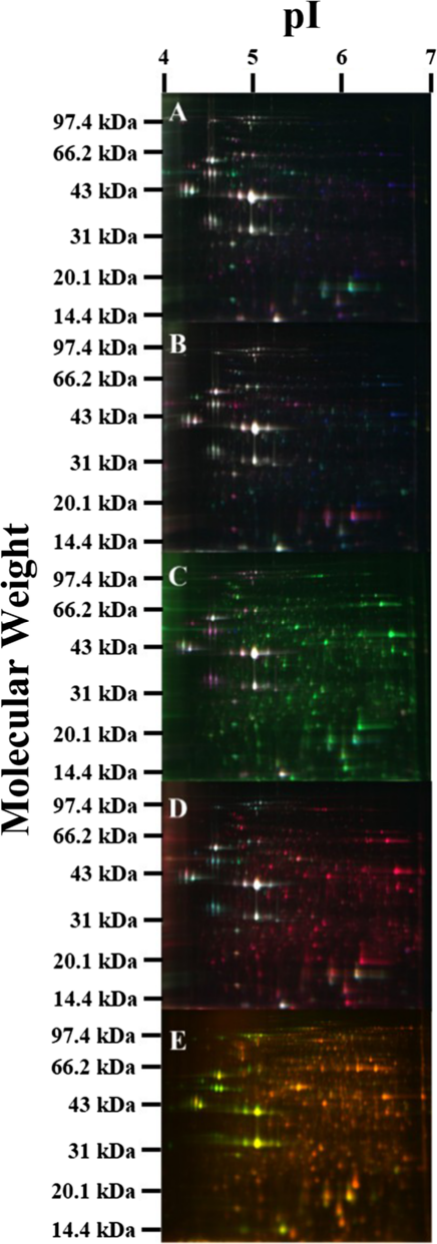
Proteomic comparison of *E. cloacae* under three different culture conditions using 2D-DIGE. Protein samples (150 μg each) from total *E. cloacae* lysates were labeled with Cy-dyes and separated using 22-cm, pH 4–7 linear IPG strips. Note: A-gel1, B-gel2, C-gel3, D-gel4, E-gel5

**Fig. 2:**
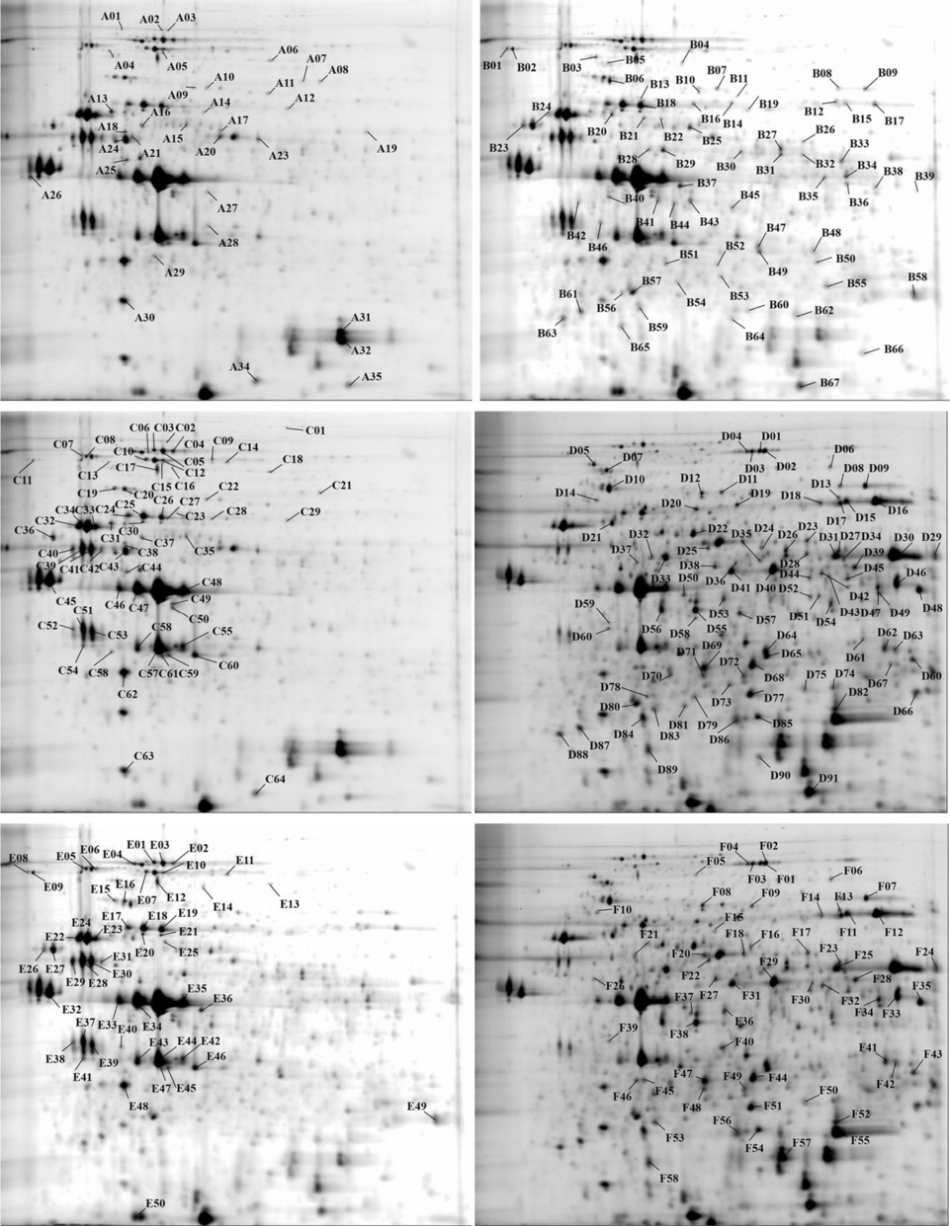
Proteins exhibiting significant changes were selected for MS/MS identification. A and B denote the upregulated proteins in Y1 and Y2 and in Y1 compared with Y2, respectively. C and D denote the upregulated proteins in Y1 and Y3 and in Y1 compared with Y3, respectively. E and F denote the up-regulated proteins in Y2 and Y3 and in Y2 compared with Y3, respectively

2D-DIGE was applied to analyze the changes in the proteome of *E. cloacae* under the different culture conditions. The gel images from the 2D-DIGE separation of *E. cloacae* are presented in Fig. 1. An average of 1,700 spots were detected in all five 2D-DIGE gels; 720 spots were reproducibly matched to all samples (triplicate runs), and the protein regulatory conditions and the success rate of detection by MS/MS are listed in Table 2. Among the three samples from the three culture conditions, changes greater than 1.5-fold and *p*-values < 0.05 were considered significant changes in protein abundance. The regulated proteins were selected for identification by MS/MS (Fig. 2), and Additional file 3 shows the MS/MS-identified proteins from *E. cloacae* cultured under three different conditions. A total of 109 types of protein were successfully identified by MS/MS, which identified 3 or more unique peptides with a confidence of 95 % at the protein level and 99 % at the peptide level.

**Table 2:** The condition of the different expression of proteins and the detection of MS/MS in *E. cloacae* cultured in three different media

Group	Y1 up-regulated	Y2 up-regulated	Y3 up-regulated	Success rate
Y1:Y2	35^a^ (30)^b^	67 (35)	-	63.73 %
Y1:Y3	64 (45)	-	91 (76)	78.06 %
Y2:Y3	-	50 (28)	58 (49)	71.30 %

^a^The number of upregulated proteins. ^b^The number of protein which detected successfully by MS/MS

### GO annotation

**Fig. 3:**
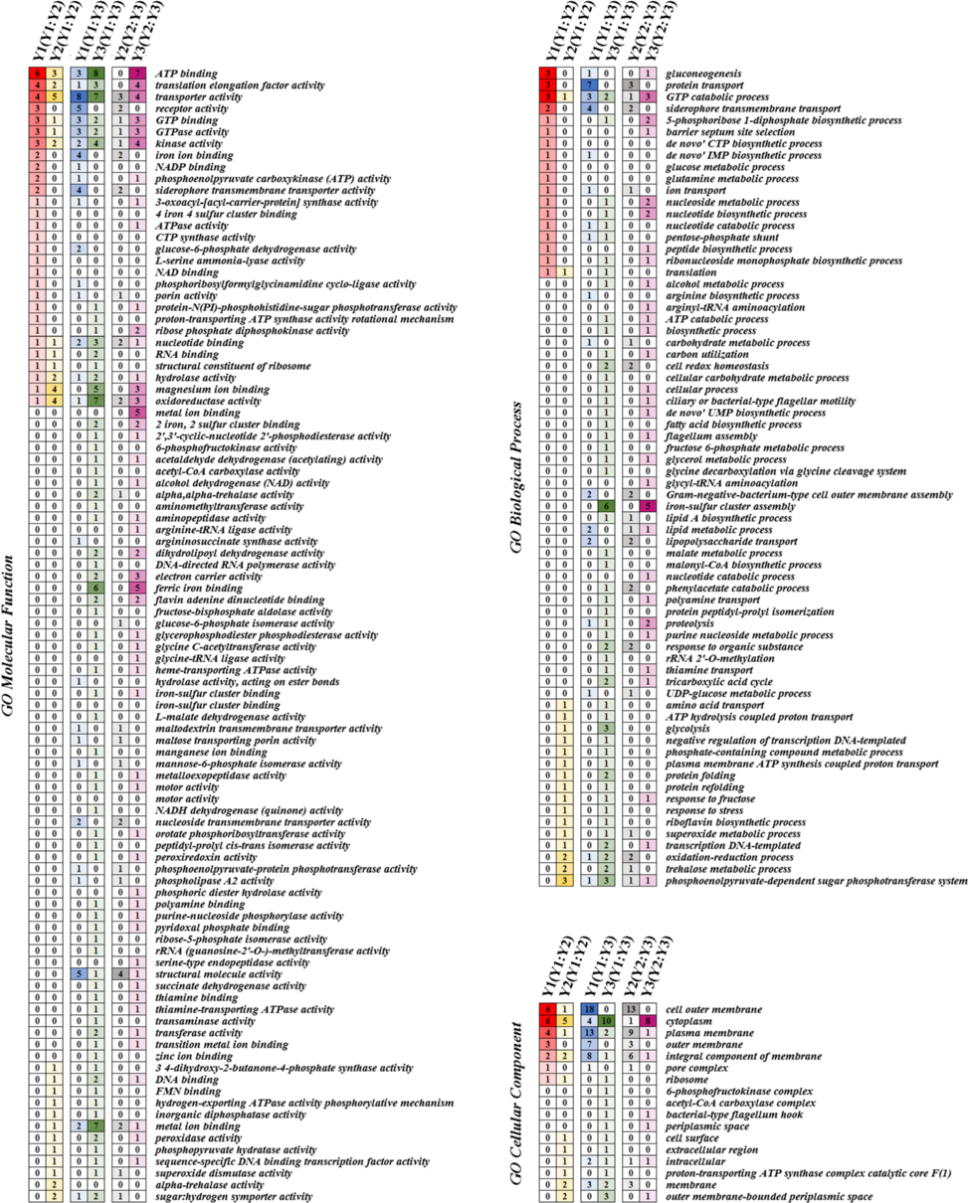
GO categorization of differentially expressed proteins in *E. cloacae* cultured under three different conditions. The proteins were classified as follows: cellular component, molecular function, and biological processes, according to the GO terms. The color of the square is related to the number of times each function was clustered by proteins; a higher frequency is represented by richer shades of each respective color

To understand the biological functions of the differentially expressed proteins under the three treatment conditions, GO annotation was performed. GO representation of the *E. cloacae* clusters was categorized according to the biological process, cellular component and molecular function (Fig. 3). Each identified protein was classified according to its GO functional annotation. These differentially expressed proteins were mainly localized in the cellular outer membrane, cytoplasm, and plasma membrane and to participate in ATP binding, protein transport and transporter activity.

**Fig. 4:**
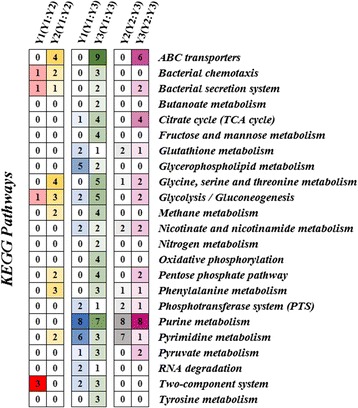
Details of the pathways that cluster with the differentially expressed proteins in the three groups. The color of the square is related to the number of regulated proteins clustered in the pathways; richer shades of each respective color indicate a higher number

According to the KEGG metabolic pathway maps of *E. cloacae*, a total of 23 pathways were clustered into three groups together with the differentially expressed proteins (Fig. 4). Among them, the ABC transporters, citric acid cycle (TCA cycle), glycerophospholipid metabolism, purine metabolism and pyrimidine metabolism were the pathways that were most influenced by the differentially expressed proteins.

### RT-PCR analysis of differentially expressed proteins

Twenty-seven genes corresponding to the protein spots that were highly differentially expressed, or related to pathogenicity, were selected for RT-PCR analysis to validate their transcript levels. The relationship between the level of protein and mRNA is displayed in Additional file 4. The RT-PCR results were consistent with those of the DIGE studies and suggested that some proteins that were identified as differentially abundant were regulated at the transcriptional level (positive correlation), such as the expression of F0F1 ATP synthase subunit beta (B20), whereas others were not (negative correlation), including the type VI secretion system protein ImpC (A11). Furthermore, some proteins showed no significant correlation between the expression of protein and the gene, such as outer membrane channel protein (A15).

### ^1^H NMR spectroscopic analysis of metabolites of E. cloacae

**Fig. 5:**
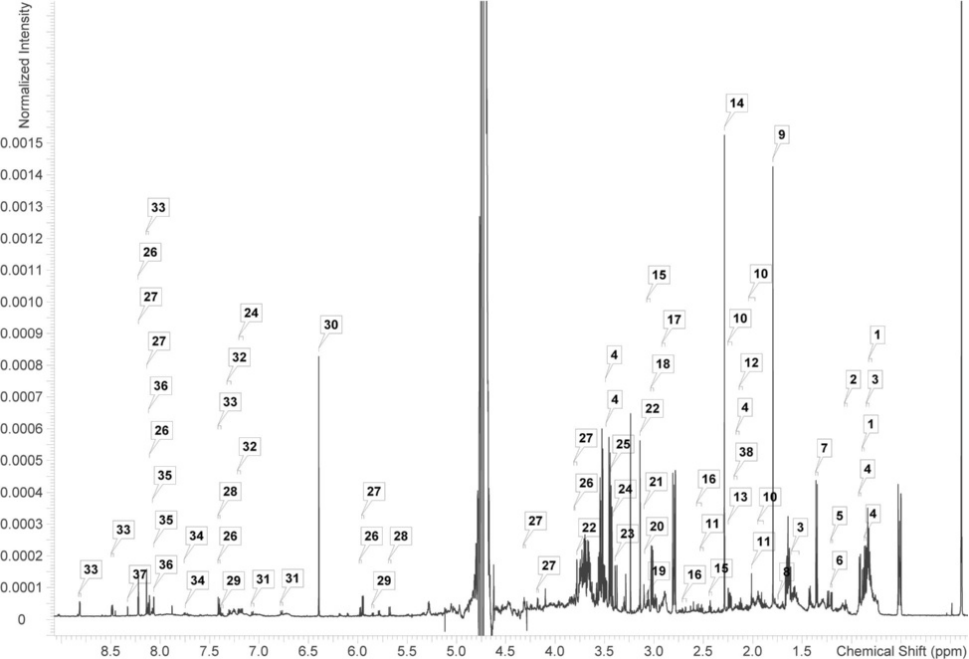
Typical 600 MHz 1H NMR spectra of *E. cloacae* extract. 1: isoleucine; 2: ethanol; 3: leucine; 4: valine; 5: threonine; 6: lactate; 7: alanine; 8: thymine; 9: acetate; 10: glutamate; 11: methionine; 12: 2-aminoadipate; 13: pyruvate; 14: succinate; 15: 2-alanine; 16: aspartate; 17: lysine; 18: ethanolamine; 19: choline; 20: O-phosphocholine; 21: sn-glycero-3-phosphocholine; 22: betaine; 23: 3-methylxanthine; 24: phenylacetate; 25: glycine; 26: inosine; 27: adenosine; 28: uracil; 29: cytosine; 30: fumarate; 31: tyrosine; 32: phenylalanine; 33: nicotinate; 34: uridine; 35: hypoxanthine; 36: adenine; 37: formate; 38: 4-aminobutyrate

**Fig. 6:**
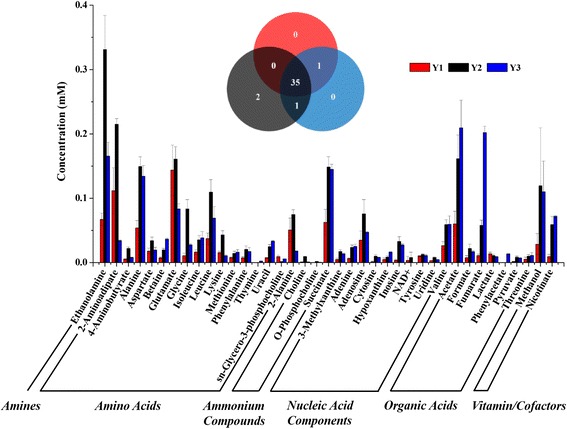
Venn diagram and histogram of the quantified levels of metabolites detected in *E. cloacae* under the three different treatment conditions

The ^1^H NMR spectra revealed several metabolites that were modified in *E. cloacae* stimulated by the blood agar plate and Fe (Figs. 5 and 6). A total of 38 individual metabolites were detected in the three treatment groups. Among the 38 types of metabolites, 35 were detected in all treatment groups, thymine and phenylacetate were only detected in normal culture, and NAD^+^ was not detected in the control group. Additionally, O-phosphocholine was not detected in the blood agar plate culture.

**Fig. 7:**
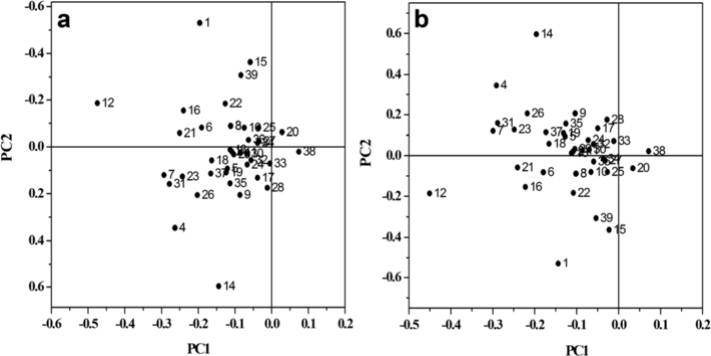
PCA (**a**) and PLS-DA (**b**) score plot illustrating the variance between 38 metabolites under the three treatment conditions applied to *E. cloacae*. 1: 2-aminoadipate, 2: 3-methylxanthine, 3: 4-aminobutyrate, 4: acetate, 5: adenine, 6: adenosine, 7: alanine, 8: aspartate, 9: betaine, 9: choline, 10: cytosine, 11: ethanolamine,12: formate, 13: fumarate, 14: glutamate, 15: glycine, 16: hypoxanthine, 17: inosine, 18: isoleucine, 19: lactate, 20: leucine, 21: lysine, 22: methanol, 23: methionine, 24: NAD+, 25: nicotinate, 26: O-phosphocholine, 27: phenylacetate, 28: phenylalanine, 29: pyruvate, 30: succinate, 31: threonine, 32: thymine, 33: tyrosine, 34: uracil, 35: uridine, 36: valine, 37: sn-glycero-3-phosphocholine, 38: 2-alanine

Three treatments effects on *E. cloacae* metabolites were emphasized during PCA and PLS-DA (Fig. 7). The PCA (principal component analysis) and PLS-DA (partial least squares discriminant analysis) showed that fumarate, acetate, ethanolamine, 2-aminoadipate, glutamate, 2-alanine, glycine, alanine and succinate made an important contribution to distinguishing among the three samples.

### Comparison of pathways affected by differentially expressed proteins by the differentially expressed metabolites

The metabolome adds an additional level of information in biological systems that reflects phenotypic and functional variation. Metabolites identified by ^1^H NMR were used to verify the pathways affected by the differentially expressed proteins. First, we classified the pathways into those that were dysregulated in only one treatment group, and then we selected the metabolites that displayed statistically significant changes in abundance (at least 1.5-fold, *p* < 0.05) (Tables 3, 4, 5). Most pathways were verified by the different metabolites, such as in Y1:Y2, and ABC transporters were regulated by proteins that were upregulated in Y2. Eleven metabolites were associated with this pathway, in which 9 compounds (alanine, betaine, glycine, isoleucine, leucine, methionine, phenylalanine, valine, threonine) had a high abundance in the Y2 treatment group and 2 compounds (glutamate, 2-alanine) were highly expressed in the Y1 treatment group.

**Table 3:** Validation of pathways affected by in difference expression protein by the different metabolic detected by ^1^H NMR (Y1 compared with Y2)

The condition of regulated by proteins	Pathways	Upregulated Metabolites	
		Y1	Y2
Y1 upregulated	Two-component system	1^a^	2^b^
Y2 upregulated	ABC transporters	2^c^	9^d^
	Glycine, serine and threonine metabolism	-	4^g^
	Methane metabolism	-	5^h^
	Pentose phosphate pathway	-	1^l^
	Phenylalanine metabolism	-	4^k^
	Pyrimidine metabolism	1^e^	5^f^
Y1 and Y2 Co-regulated	Bacterial chemotaxis	-	-
	Bacterial secretion system	-	-
	Glycolysis / Gluconeogenesis	1^i^	2^j^

^a^Glutamate. ^b^Succinate, Fumarate. ^c^Glutamate, 2-Alanine. ^d^Alanine, Betaine, Glycine, Isoleucine, Leucine, Methionine, Phenylalanine, Valine, Threonine. ^e^2-Alanine. ^f^Alanine, Thymine, Uracil, Cytosine, Uridine. ^g^Betaine, Glycine, Pyruvate, Threonine. ^h^Glycine, Acetate, Formate, Pyruvate, Methanol. ^i^Lactate. ^j^Acetate, Pyruvate. ^k^Phenylalanine, Succinate, Fumarate, Pyruvate. ^l^Pyruvate

**Table 4:** Verify pathways affected by in difference expression protein by the different metabolic detected by ^1^H NMR (Y1 compared with Y3)

The condition of regulated by proteins	Pathways	Upregulated Metabolites	
		Y1	Y3
Y3 upregulated	Bacterial secretion system	-	-
	Butanoate metabolism	-	3^a^
	Nitrogen metabolism	-	1^b^
	Bacterial chemotaxis	-	1^c^
	Phenylalanine metabolism	-	4^d^
	Tyrosine metabolism	-	2^e^
	Fructose and mannose metabolism	-	-
	Methane metabolism	-	5^f^
	Oxidative phosphorylation	-	3^g^
	Pentose phosphate pathway	-	1^h^
	Glycine, serine and threonine metabolism	-	6^i^
	ABC transporters	-	13^j^
Y1 and Y3 Co-regulated	Pyruvate metabolism	-	5^k^
	Citrate cycle	-	3^l^
	Glutathione metabolism	-	1^m^
	Phosphotransferase system	-	1^n^
	RNA degradation	-	-
	Nicotinate and nicotinamide metabolism	-	5°
	Two-component system	-	3^p^
	Glycolysis/Gluconeogenesis	-	2^q^
	Glycerophospholipid metabolism	1^r^	2^s^
	Pyrimidine metabolism	-	4^t^
	Purine metabolism	-	5^u^

^a^Succinate, Fumarate, Pyruvate. ^b^Formate. ^c^Aspartate. ^d^Phenylalanine, Succinate, Fumarate, Pyruvate. ^e^Fumarate, Pyruvate. ^f^Glycine, Acetate, Formate, Pyruvate, Methanol. ^g^Succinate, NAD^+^, Fumarate. ^h^Pyruvate. ^i^Aspartate, Betaine, Choline, Glycine, Pyruvate, Threonine. ^j^Alanine, Aspartate, Betaine, Glycine, Isoleucine, Leucine, Lysine, Methionine, Phenylalanine, Choline, Succinate, Valine, Threonine. ^k^Succinate, Acetate, Formate, Fumarate, Pyruvate. ^l^Succinate, Fumarate, Pyruvate. ^m^Glycine. ^n^Pyruvate. ^o^Aspartate, NAD^+^, Fumarate, Pyruvate, Nicotinate. ^p^Aspartate, Succinate, Fumarate. ^q^Acetate, Pyruvate. ^r^sn-Glycero-3-phosphocholine.^s^Ethanolamine, Choline. ^t^Alanine, Uracil, Cytosine, Uridine. ^u^Glycine, Adenine, Adenosine, Hypoxanthine, Inosine

**Table 5:** Verify pathways affected by in difference expression protein by the different metabolic detected by ^1^H NMR (Y2 compared with Y3)

The condition of regulated by proteins	Pathways	Upregulated Metabolites	
		Y2	Y3
Y3 upregulated	Bacterial secretion system	-	-
	Glycolysis/Gluconeogenesis	-	-
	Pentose phosphate pathway	-	-
	Pyruvate metabolism	-	1^a^
	Citrate cycle	-	1^b^
	ABC transporters	1^c^	6^d^
Y2 and Y3 Co-regulated	Phenylalanine metabolism	1^e^	-
	Glycine, serine and threonine metabolism	1^f^	3^g^
	Glutathione metabolism	-	2^h^
	Phosphotransferase system	-	-
	Nicotinate and nicotinamide metabolism	-	-
	Pyrimidine metabolism	-	-
	Purine metabolism	-	-

^a^Fumarate. ^b^Fumarate. ^c^Betaine. ^d^Aspartate, Glutamate, Glycine, Leucine, Lysine, Choline. ^e^Fumarate. ^f^Betaine. ^g^Aspartate, Glycine, Choline. ^h^Glutamate, Glycine

## Discussion

### Proteins involved in iron uptake and utilization

Transport proteins play an important role in pathogenicity. This class includes toxins, trans-envelope protein secretion systems, outer membrane protein secretion systems and outer membrane iron-siderophore receptors that function with cytoplasmic membrane ABC-type iron uptake transporters [16]. In our limited research, we were interested in investigating transport proteins related to iron absorption and transportation, and in correlating them with pathogenicity.

In the comparison of Y1 and Y2, the ferrichrome outer membrane transporter (A02, A05), L-serine ammonia-lyase (A14), and hypothetical protein EcWSU1_01016 (A08) (GO cluster analysis associated this protein with metal ion binding (GO:0046872)) were upregulated in *E. cloacae* cultured on a blood agar plate. The sheep blood used in this plate provided the iron ions required by *E. cloacae*, improving its pathogenicity.

In Y1 compared with Y3, the upregulated proteins linking iron absorption and transportation in Y2 were ferric aerobactin receptor (C08), ferrichrome outer membrane transporter (C10, C12, C15), phosphoenolpyruvate-protein phosphotransferase (C19), hypothetical protein EcWSU1_01016 (C21) and LamB type porin (C32). The expression of these 6 proteins was up-regulated more than 5-fold in Y1 compared with Y3. In contrast, the levels of 2’,3’-cyclic-nucleotide 2’-phosphodiesterase/3’-nucleotidase (D06), maltose ABC transporter periplasmic protein (D30, D40, D41, D68, D69, D77), phenylacetate-CoA oxygenase, NAD(P)H oxidoreductase component (D31), 6-phosphofructokinase (D44), methionine aminopeptidase (D53), phenylacetic acid degradation protein paaC (D60), osmolarity response regulator (D62), bifunctional acetaldehyde-CoA/alcohol dehydrogenase (D63), succinate dehydrogenase iron-sulfur subunit (D66) and NADH-quinone oxidoreductase subunit E (D79) were up-regulated more than 5-fold in Y3 compared with Y2. The most up-regulated protein was maltose ABC transporter periplasmic protein (D30), with a more than 59-fold increase in expression. The DIGE results suggested that *E. cloacae* expresses more proteins to absorb and transport iron under iron-rich culture conditions. The growth curve of *E. cloacae* under different culture conditions and varying concentrations of Fe^3+^ revealed that high concentrations of Fe^3+^ had a certain inhibitory effect on growth. Although we selected a Fe^3+^ concentration that could promote the growth of *E. cloacae*, we hypothesize that it is difficult for the bacteria to take up and utilize iron from the blood agar plate culture. However, a continuous increase in the concentration of Fe^3+^ may inhibit growth as well as iron uptake and utilization (Additional file 1).

This speculation was confirmed in Y2 compared with Y3 group. We found that some of the previously mentioned proteins related to iron uptake and utilization were upregulated in the common Y2 culture, such as ferric aerobactin receptor (E05), ferrichrome outer membrane transporter (E07), and LamB type porin (E22), among others. In comparisons of the iron concentration, a greater content was detected in Y3 compared with Y1. These findings further suggest that the concentration of iron is important for the growth and pathogenicity of the bacteria.

The identification of the ferric uptake regulator (Fur) family was quite interesting. Fur plays a crucial role in bacterial metabolism, and iron deficiency is the most common nutritional stress during the process of cell survival [17]. In most prokaryotic organisms, Fur controls iron metabolism and plays a role in the regulation of defenses against oxidative stress. It regulates the expression of iron-binding proteins, which depend on the concentration of iron in the cell [18].

### Glycerophospholipid metabolism and ATP-binding cassette (ABC) transporters

After comparing all of the identified pathways, we observed a relationship between glycerophospholipid metabolism and ATP-binding cassette (ABC) transporters in Y1 compared with Y3. Although higher throughput protein analysis technology such as iTRAQ [19] and higher frequency NMR [20] were not used in our limited research, we still identified the relationship between the proteins and metabolites.

**Fig. 8:**
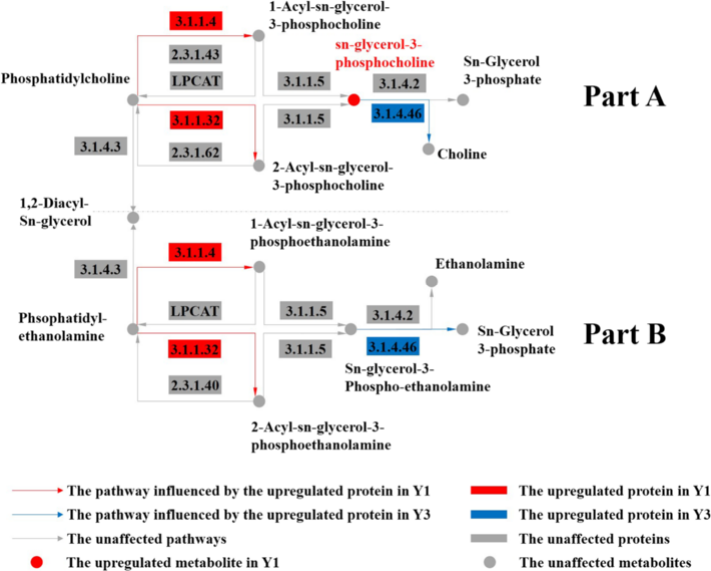
Glycerophospholipid metabolism in Y1 compared with Y3. The enzymes shown in purple were upregulated in Y1, and those shown in yellow were upregulated in Y3

First, during glycerophospholipid metabolism, we located 2 dysregulated proteins using DIGE: glycerophosphodiester phosphodiesterase (EC:3.1.4.46, F32) and phospholipase A (EC:3.1.1.4, EC:3.1.1.32, E42). Part A of Fig. 8 shows that phospholipase A can catalyze two biosynthetic processes that utilize phosphatidylcholine to synthesize 1-acyl-sn-glycero-3-phosphocholine and 2-acyl-sn-glycero-3-phosphocholine. Subsequently, lysophospholipase synthesizes sn-glycero-3-phosphocholine. In Y1, we speculate that sn-glycero-3-phosphocholine accumulated because of the increased expression level of phospholipase A and the down-regulation of glycerophosphodiester phosphodiesterase. The metabolomic results corroborated this hypothesis because the concentration of sn-glycero-3-phosphocholine in Y1 was up-regulated more than 6-fold compared with Y3.

In part B of the glycerophospholipid metabolism analysis (Fig. 8), although glycerophosphodiester phosphodiesterase was highly expressed, we did not detect a difference in sn-glycerol 3-phosphate between the Y1 and Y3 treatment groups. After searching the pathways associated with sn-glycerol 3-phosphate, we found that sn-glycerol 3-phosphate also belongs to the ABC transporter family. After sequencing the genome of *Edwardsiella tarda* EIB202, Wang et al. identified and localized a large number of ABC system components. The ETAE_0613 and ETAE_0907 components of the ABC system are potential virulence genes, which may provide insight into the relationship between the output of virulence factors and antibiotics and the acquisition of sn-glycerol 3-phosphate [21].

The ATP-binding cassette (ABC) transporters form one of the largest known protein families and are widespread in bacteria, archaea, and eukaryotes. They couple ATP hydrolysis to the active transport of a wide variety of substrates such as ions, sugars, lipids, sterols, peptides, proteins, and drugs. ABC transporters are dedicated to the export of virulence factors under appropriate conditions such as our iron-rich culture condition. An example is provided by iron ABC uptake systems, which have long been recognized as important effectors of virulence [22]. Because iron exists primarily in the insoluble Fe^3+^ form under aerobic conditions, biologically available iron in the body is found chelated by high-affinity iron-binding proteins (BPs) (e.g., transferrins, lactoferrins, and ferritins) or as a component of erythrocytes (such as heme, hemoglobin, or hemopexin) [23]. Pathogens are able to scavenge iron from these sources by secreting high affinity iron-complexing molecules called siderophores and reabsorbing them as iron-siderophore complexes [24]. For example, lactoferrin-binding protein B (LbpB) is a bi-lobed membrane-bound lipoprotein that is part of the lactoferrin receptor complex in a variety of Gram-negative pathogens [25]. Our DIGE results revealed the location of the iron complex transport system based on the differential expression of the iron-hydroxamate transporter ATP-binding subunit (EC: 3.6.3.34, F41) (using DIGE, F41 were identified as an osmolarity response regulator by MS/MS, after transformation in the *E. cloacae* subsp. cloacae ENHKU01 by BLAST, the spot was confirmed to be iron-hydroxamate transporter ATP-binding subunit, with 95 % confidence). After comparing the expression of this protein among the three treatment groups, the abundance was ranked as Y3 > Y1 > Y2. This result indicated that Fe stimulation of *E. cloacae* was greater in the medium with Fe supplementation than in the blood agar plate.

### Bacterial secretion system

The host interactions of pathogenic bacteria are usually mediated via protein secretion mechanisms. Gram-negative pathogenic bacteria will transport protein to the extracellular environment or to the host cell through devices called secretion systems. To date, a least six different types of secretion systems have been discovered in Gram-negative pathogenic bacteria (I-VI secretion system). These systems can stimulate and interfere with the processes of host cells by secreting or releasing and injecting extracellular proteins or effectors [26]. Using DIGE, we located two types of secretion systems, type I and VI, based on the differential expression of the outer membrane channel protein (TolC), type VI secretion system secreted protein Hcp (hemolysin co-regulated protein) and type VI secretion system protein ImpC.

The type I secretion systems (T1SS) are responsible for the release of a variety of extracellular proteins and extracellular enzymes [27]. TolC, which we identified by DIGE, is associated with multiple drug resistance in bacteria. The expression of TolC in the three treatment groups was as follows: Y1 > Y2 > Y3. Although the majority of research investigating type I secretion systems has focused on multiple drug resistance, it can be speculated from the results of our study that the protein also has an association with pathogenicity and participates in responses to differences in iron stimulation.

In contrast, type VI secretion systems (T6SS) have a clear and strong correlation to pathogenicity, and nearly all confirmed functional T6SS are poisonous to macrophages [28]. Hcp can cross the T6SS transport channel to enter the plasma and interact with the host through the help of lipoprotein [29]. Due to protein modifications or degradation, as mentioned previously, in the group of Y1 compared with Y2, similar Hcp levels were observed. In contrast, in Y1 compared with Y3, the expression of Hcp in Y3 was almost 10-fold higher than that in Y1. Similarly, the type VI secretion system protein ImpC was approximately 2-fold higher in Y3 than in Y2. These findings indicated that the secretion of Hcp was regulated by iron, which supports the research of Wang et al., who found that iron was one of the regulators of the T6SS component *evp*P in *Edwardsiella tarda* [30].

### Two-component system

Two-component signal transduction systems enable bacteria to sense, respond, and adapt to changes in their environment or in their intracellular environment. In this experiment, differences in the two-component system were observed between Y1 and Y2 and between Y1 and Y3, but not between Y2 and Y3. The differentially expressed proteins were involved in resistance to the osmotic upshift (K^+^). This result was consistent with our experimental design, and the major difference between the Y2 and Y3 culture conditions was the presence of iron; however, in Y1, the blood agar plate culture condition was the main difference compared with Y2 and Y3, in which the sheep blood fiber is enriched with a variety of elements, including K^+^. Bacteria are sensitive to changes in the external environment, and consequently they undergo a series of mechanisms to adapt and protect themselves. Specifically, they must protect themselves against the immune response of the host during infection. In the blood agar plate condition, K^+^ potentially caused a change in osmotic pressure, stressing the cells and potentially leading to cell lysis and death. As a result, the two-component system probably helped the bacteria resist the change in osmotic pressure and protected them by regulating the expression of outer membrane proteins. Although the system has mainly been reported in terms of bacterial responses to climate change, the present study also shows that the system plays a role in pathogenicity. The system can adjust the various metabolic processes of bacteria, the bacterial cell cycle, the exchange of signals between bacteria and the expression of virulence factors [31].

## Conclusion

The regulation of environmental factors leads to both physiological and biochemical changes in bacteria. As a result, the pathogenicity of the bacteria also changes in response to environmental stimuli. The results of this study showed that the blood agar plate and a suitable concentration of iron ions enhanced the pathogenicity of *E. cloacae* and that very high concentrations of iron may have had an adverse effects on growth and on iron uptake and utilization by this bacteria. It is difficult to make an absolute comparison of the stimulatory effect of blood versus iron on pathogenicity. The pathogenicity of *E. cloacae* is affected by their living conditions and the condition of the bacteria.

## Additional files

Additional file 1: Growth curve of *E. cloacae* cultured under six different Fe^3+^ concentrations. (PDF 116 kb)
Additional file 2: Primer sequence for RT-PCR (XLS 28 kb)
Additional file 3: Differentially expressed protiens upon three treatment as identified by MALDI-TOF-MS/MS (XLS 77 kb)
Additional file 4: The correlation of expression between protiens and genes (XLS 40 kb)

## Abbreviations

2D-DIGE: Two-dimensional difference gel electrophoresis
ABC: ATP-binding cassette
ACDSS: Anachro-Certified DSS Standard Solution
DSS-d6: 2, 2-dimethyl-2-silapentane-5-sulfonate-d6 sodium salt
EC: Enzyme commission
ESBLs: Extended-spectrum β-lactamases
Fur: Ferric uptake regulator
GO: Gene ontology
Hcp: Hemolysin co-regulated protein
IEF: Iso-electric focusing
IPG: Immobilized pH gradients
Itraq: Isobaric tags for relative and absolute quantitation
NMR: Nuclear magnetic resonance
PBS: Phosphate-buffered saline
RT-PCR: Reverse transcription PCR
SDS-PAGE: Sodium dodecyl sulfate-polyacrylamide gel electrophoresis
T1SS: Type I secretion systems
T6SS: Type VI secretion systems

## References

[1] Cascio A, Mezzatesta ML, Odierna A, Di Bernardo F, Barberi G, Iaria C, Stefani S, Giordano S (2014). Extended-spectrum beta-lactamase-producing and carbapenemase-producing *Enterobacter cloacae* ventriculitis successfully treated with intraventricular colistin. Int J Infect Dis.

[2] Dijk Y, Bik E, Hochstenbach-Vernooij S, Vlist G, Savelkoul P, Kaan J, Diepersloot R (2002). Management of an outbreak of *Enterobacter cloacae* in a neonatal unit using simple preventive measures. J Hosp Infect.

[3] Sanders W, Sanders CC (1997). *Enterobacter* spp.: pathogens poised to flourish at the turn of the century. Clin Microbiol Rev.

[4] Hoffmann H, Roggenkamp A (2003). Population genetics of the nomenspecies Enterobacter cloacae. Appl Environ Microbiol.

[5] Yu W-L, Cheng H-S, Lin H-C, Peng C-T, Tsai C-H (2000). Outbreak investigation of nosocomial *Enterobacter cloacae* bacteraemia in a neonatal intensive care unit. Scand J Infect Dis.

[6] Antony B, Prasad BPMR (2011). An outbreak of neonatal septicaemia by *Enterobacter cloacae*. Asian Pac J Trop Dis..

[7] Zhou Q, Zhang M, Wang A, Xu J, Yuan Y (2012). Eight-Year Surveillance of Antimicrobial Resistance among *Enterobacter Cloacae* Isolated in the First Bethune Hospital. Phys Procedia.

[8] Yogaraj JS, Elward AM, Fraser VJ (2002). Rate, risk factors, and outcomes of nosocomial primary bloodstream infection in pediatric intensive care unit patients. Pediatrics.

[9] Zhang D, Li C, Zhou J, Zhang C, Wang Z, Su X (2013). Research of the structure diversity of bacteria form Ningbo coastal outfall and virulence genes associated with iron metabolism. Oceanologia et limnologia sinica.

[10] Lu F, Miao S, Tu J, Ni X, Xing L, Yu H, Pan L, Hu Q (2013). The role of TonB-dependent receptor TbdR1 in Riemerella anatipestifer in iron acquisition and virulence. Vet Microbiol.

[11] Olakanmi O, Kesavalu B, Abdalla MY, Britigan BE (2013). Iron acquisition by Mycobacterium tuberculosis residing within myeloid dendritic cells. Microb Pathog.

[12] Braun V (2001). Iron uptake mechanisms and their regulation in pathogenic bacteria. Int. J. Med. Microbiol..

[13] Brown JS, Holden DW (2002). Iron acquisition by Gram-positive bacterial pathogens. Microbes Infect.

[14] Mahapatra A, Ghosh S, Mishra S, Pattnaik D, Pattnaik K, Mohanty S (2002). *Enterobacter cloacae*: a predominant pathogen in neonatal septicaemia. Indian J Med Microbiol.

[15] Daniels NA, Shafaie A (2000). Review of pathogenic Vibrio infections for clinicians. Infect Med.

[16] Tang F, Saier MH. Transport proteins promoting *Escherichia coli* pathogenesis. Microb Pathog. 2014;71(1):41–55. 10.1016/j.micpath.2014.03.008 PMC4106412 24747185

[17] Fillat MF (2014). The FUR (ferric uptake regulator) superfamily: diversity and versatility of key transcriptional regulators. Arch Biochem Biophys.

[18] Wee S, Neilands JB, Bittner ML, Hemming BC, Haymore BL, Seetharam R (1988). Expression, isolation and properties of Fur (ferric uptake regulation) protein of *Escherichia coli* K 12. Biol Met.

[19] Kaltwasser B, Schulenborg T, Beck F, Klotz M, Schafer KH, Schmitt M, Sickmann A, Friauf E (2013). Developmental changes of the protein repertoire in the rat auditory brainstem: a comparative proteomics approach in the superior olivary complex and the inferior colliculus with DIGE and iTRAQ. J Proteomics.

[20] Masetti O, Ciampa A, Nisini L, Valentini M, Sequi P, Dell’Abate MT (2014). Cherry tomatoes metabolic profile determined by ^1^H-High Resolution-NMR spectroscopy as influenced by growing season. Food Chem.

[21] Wang Q, Yang M, Xiao J, Wu H, Wang X, Lv Y, Xu L, Zheng H, Wang S, Zhao G (2009). Genome sequence of the versatile fish pathogen *Edwardsiella tarda* provides insights into its adaptation to broad host ranges and intracellular niches. PLoS One.

[22] Henderson DP, Payne SM (1994). *Vibrio cholerae* iron transport systems: roles of heme and siderophore iron transport in virulence and identification of a gene associated with multiple iron transport systems. Infect Immun.

[23] Köster W (2001). ABC transporter-mediated uptake of iron, siderophores, heme and vitamin B 12. Res Microbiol.

[24] Wandersman C, Delepelaire P (2004). Bacterial iron sources: from siderophores to hemophores. Annu Rev Microbiol.

[25] Morgenthau A, Beddek A, Schryvers AB (2014). The negatively charged regions of lactoferrin binding protein B, an adaptation against anti-microbial peptides. PLoS One.

[26] Yoshida Y, Miki T, Ono S, Haneda T, Ito M, Okada N (2014). Functional characterization of the type III secretion ATPase SsaN encoded by salmonella pathogenicity island 2. PLoS One.

[27] Delepelaire P (2004). Type I secretion in gram-negative bacteria. Biochim Biophys Acta.

[28] Shanks J, Burtnick MN, Brett PJ, Waag DM, Spurgers KB, Ribot WJ, Schell MA, Panchal RG, Gherardini FC, Wilkinson KD (2009). Burkholderia mallei tssM encodes a putative deubiquitinase that is secreted and expressed inside infected RAW 264.7 murine macrophages. Infect Immun.

[29] Shrivastava S, Mande SS (2008). Identification and functional characterization of gene components of Type VI Secretion system in bacterial genomes. PLoS One.

[30] Wang X, Wang Q, Xiao J, Liu Q, Wu H, Xu L, Zhang Y (2009). *Edwardsiella tarda* T6SS component evpP is regulated by esrB and iron, and plays essential roles in the invasion of fish. Fish Shellfish Immunol.

[31] Hoch JA (2000). Two-component and phosphorelay signal transduction. Curr Opin Microbiol.

