# Supplementary figures and images for: Multi-omics analysis on the pathogenicity of Enterobacter cloacae ENHKU01 isolated from sewage outfalls along the Ningbo coastline
Source: Proteome Sci. 2016 Oct 18;14:15. doi: 10.1186/s12953-016-0104-y (PMC5070189; doi:10.1186/s12953-016-0104-y)

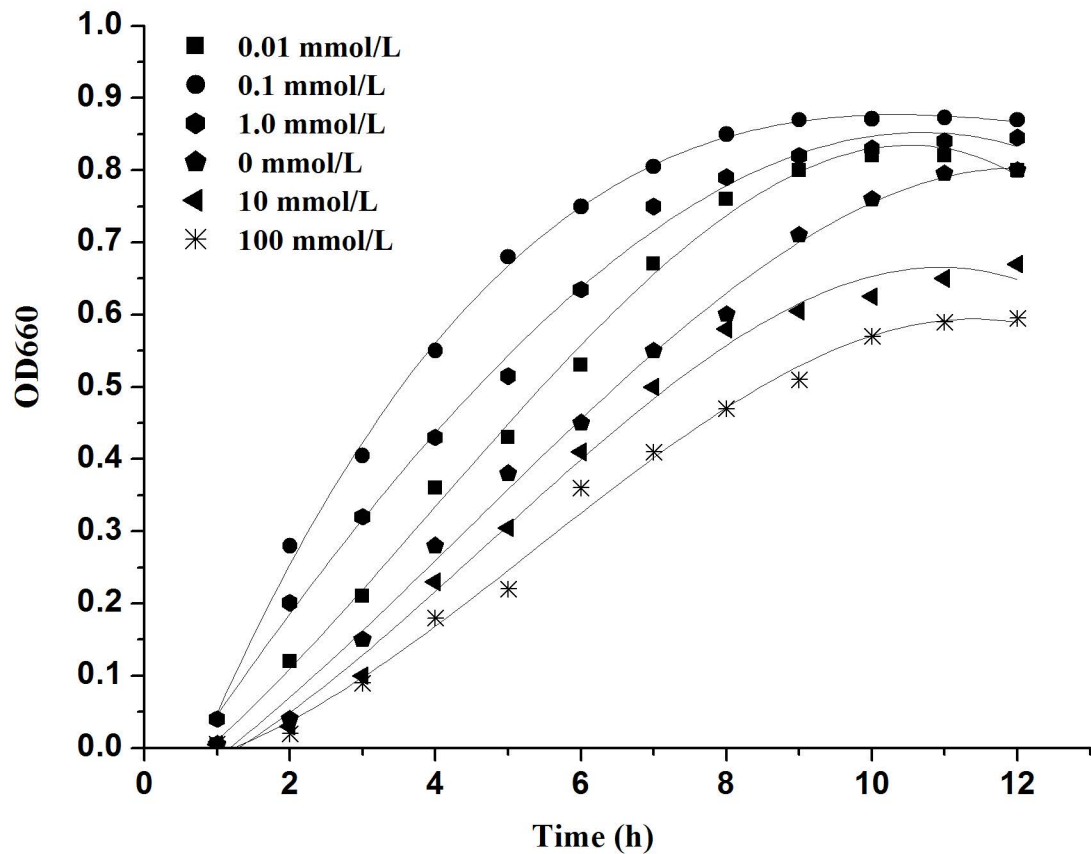

Supplement: Additional file 1: — Growth curve of E. cloacae cultured under six different Fe3+ concentrations. (PDF 116 kb) [file 12953_2016_104_MOESM1_ESM.pdf]
